# The Evolution of the “Inlay” in Dentistry

**Published:** 1907-09-15

**Authors:** F. W. MacDonald


### THE EVOLUTION OF THE "INLAY" IN DENTISTRY.

BY F. W. MACDONALD. D. D. S.

To trace the evolution of the "inlay" as we find it in the practice of dentistry today, although laborious, is to say the least interesting as well as instructive. One is taken back to the early years of operative dentistry, and though it is only in the very recent years we find the perfected inlay, yet in the very infancy of the art of dentistry we see traces here and there of the development and application of this principle of inlaying, which, I believe, is to revolutionize the present methods of filling teeth.

It is not strange that this principle of inlaying should occur to those men years ago, for it was one of the most ancient of arts and it had been brought well-nigh to perfection. The thought of restoring broken surfaces in teeth, as was being done in gold and wood was ever present with them. Conditions they know were vastly different, but they felt with a prophetic instinct that sometime and somewhere these obstacles would be removed—difficulties apparently unsurmountable would be overcome and the principle of inlaying teeth would become recognized as good practice in restoring broken down tooth tissues. First of all the problem that confronted them was that of getting a suitable material which would resemble the tooth tissue in color to a degree, and that would withstand any chemical action in the mouth. In 1820, Dr. Dinderer began the practice of using animal's teeth, grinding them down to fit the cavity, depending on the swelling of the material under moisture to make it fit accurately enough to be retained; but this method was discarded owing to the fact that these inlays soon become very much discolored, and thus the main object was defeated. From that time until 1837, practically nothing had been recorded showing the discovery of any new material. In that year, however, Doctor Murphy, of London, reported using glass in labial cavities.

In 1862 Doctor B. Wood, presented an article entitled, "Enameling Plugs and Restoring the Contour of Defective Teeth by the Application of Enameled Caps." He says: "The design of the improvement is to restore the form and beauty of decayed and broken teeth, at the same time preserving as much of the healthy dentine, and concealing the metallic plugs by means of a cap or covering resembling the sound parts of the teeth. * * * These caps are from the size of a medium sized plug to that of the entire crown of the tooth. They are made according to circumstances, with grooves, slots, cavities, orifices, serrations and with asperities made by means of platina scraps at the basis in order to retain the filling. * * * I have assumed the plastic metallic filling is the material to be used for engrafting the cap to the natural base, but it will occur that any plastic material with sufficient tenacity and otherwise suitable to the purpose could be used."

Establishing the originality of certain constructions of processes is made difficult owing to the fact that articles in the earlier days of dental journalism were not so elaborately illustrated as at the present time, and consequently we can depend only on the text. In the papers I have taken extracts from there can be no misinterpretation as to what is meant, so we may safely say that in these men we haven't the pioneers of the "inlay" in dentistry.

Doctors Linderer, Volck and Maynard endeavored to place materials in a cavity by grinding them down to fit the cavity as accurately as possible. They endeavored to get a material that resembled the natural tooth. The esthetic value of the inlay is what appealed greatly to them. They did not consider the fact that their method would save the tooth better than any other method for there is no doubt that it would not, but they were willing to sacrifice the permanency of the inlay for the improved appearance. Doctor Wood does not explain his method, but simply gives us a view of his completed work. How he attained these results we are not sure, but from the text he had some measurement which allowed him to do such work as he outlines. He is the first one to record the use of a plastic mass in setting plugs or inlays and caps and also the first one to suggest the roughening of the under surface of the inlay to make it the more secure in its attachment. He not only is the originator of the metal plug or inlay but he is the first one recorded who suggested the enameling of the outer surface of the plug or inlay as well as the metallic cap for badly broken down teeth. This to my mind is nothing more or less than the basis for our porcelain jacket crown. True there have been many modifications of this method since—changes in method of construction every year, but the fact stands that according to the records in dental journals—though not in the United States Patent Gazette, here is the man whom the dental profession is indebted for blazing the way in this branch of dental art. Following closely upon Dr. Wood's paper, Doctor Starr, in 1870, reports having had porcelain pieces in which pins were fused for the purpose of retention and Doctor Weagant had instruments constructed which would cut the cavity to correspond to the same sized piece of porcelain. In 1876, Doctor E. A. Bogue, is reported as saying, "Doctor Williams, of Boston, sent me two or three little toadstools in shape requesting that I exhibit them. They are the device of Doctor Fisk, I think of Massachusetts, who has used them in filling teeth with gutta percha, then warming the gold and pressing it home. The gold is somewhat the shape of an umbrella, the tent of the umbrella being shaped as the surface of a large gold filling would be, so that you have a gutta percha filling and a gold cap as a protector to the gutta percha. The gold is retained by a pivot, pin or post which was previously attached to its under surface and which engages with the plastic material.

From this time on modifications of the methods previously mentioned were recorded every year. M. H. Webb, in November and December number of the Dental Cosmos, of 1879, and in the May number of 1883, Doctor S. D. Rambo, in the Dental Cosmos of 1882, Doctor C. H. Land, Patent

Gazette, December, 1887, and A. H. Thompson in the Western Dental Journal of May, 1888, all record original modifications.

Doctor C. H. Land's patent covering the method of burnishing ribbon platinum into the cavity, making a matrix the same form as the cavity and fusing porcelain in it with the aid of a gas furnace was executed in December, 1887. It undoubtedly was distinctly different from any other method recorded up till that time as far as we can determine, and it is this method, with modification, that is largely used in practice today in making porcelain inlays.

Doctor W. H. Jackson, of Ann Arbor, as early as 1881, used gold inlays for the purpose of restoring contours which is practically the same method as is in use today. In fact Doctor Jackson can well lay claim, as far as record goes, to the devising of the method of burnishing metal into cavities, in order to get a metallic matrix; and while Doctor Lan's patent technically gives him the credit, yet the principle of the method involved is the same as that of Doctor Jackson. His method is that of burnishing thin gold in to the prepared cavity, and after outlining the cavity, filling up with solder to the required contour, and cementing the soldered piece into the cavity.

In Dental Cosmos of 1891, page 79, Doctor Swasey gave his method of making gold inlays. He first fills all the under cuts with wax and takes impression in modelling compounds. Plaster model is then run and Melottes metal die is stuck and pure gold plate of 50 gauge is swaged and final burnishing is made in the cavity. The matrix is invested and 20 K. gold is fused in it in small pieces. Doctor G. V. I. Brown, in Cosmos of 1893, records his method of making a hollow gold inlay by filling cavity one-third full of wax before taking impression. In the same volume, page 196, Doctor Alexander records a method very similar to one being exploited at the present time. He burnishes platinum into the cavity, carves up in wax part to be restored and then burnishing platinum or gold over that, leaving "a suitable part of the wax remaining uncovered." All the metal part is invested and wax is boiled out and solder is flowed into the metal lined matrix.

This practically brings us up to the present day inlay methods. I have endeavored to ferret out those to whom originality of the different methods and modifications belong. It is surprising that many times methods will be recorded in Society proceedings, when the one who is demonstrating it either enthusiastically lays claim to its origin or developement or else passively allows others to think he is the one entitled to credit. It may be that I have given credit to some one to whom credit does not belong, but I have searched records of dental journals as best I could, to make this history short of the evolution of inlays as complete and as authentic as possible.—*Odontoblast.*

